# Valid and reliable instruments for arm-hand assessment at ICF activity level in persons with hemiplegia: a systematic review

**DOI:** 10.1186/1471-2377-12-21

**Published:** 2012-04-12

**Authors:** Ryanne JM Lemmens, Annick AA Timmermans, Yvonne JM Janssen-Potten, Rob JEM Smeets, Henk AM Seelen

**Affiliations:** 1 Research School CAPHRI, Department of Rehabilitation Medicine, Maastricht University, Maastricht, the Netherlands; 2 Adelante, Centre of Expertise in Rehabilitation and Audiology, Hoensbroek, the Netherlands; 3 Department of Rehabilitation Medicine, Maastricht University Medical Centre, Maastricht, the Netherlands

**Keywords:** Rehabilitation, Stroke, Cerebral Palsy, Arm, Hand, Outcome assessment, Activities of daily living, Activity, Capacity, Performance

## Abstract

**Background:**

Loss of arm-hand performance due to a hemiparesis as a result of stroke or cerebral palsy (CP), leads to large problems in daily life of these patients. Assessment of arm-hand performance is important in both clinical practice and research. To gain more insight in e.g. effectiveness of common therapies for different patient populations with similar clinical characteristics, consensus regarding the choice and use of outcome measures is paramount. To guide this choice, an overview of available instruments is necessary. The aim of this systematic review is to identify, evaluate and categorize instruments, reported to be valid and reliable, assessing arm-hand performance at the ICF activity level in patients with stroke or cerebral palsy.

**Methods:**

A systematic literature search was performed to identify articles containing instruments assessing arm-hand skilled performance in patients with stroke or cerebral palsy. Instruments were identified and divided into the categories capacity, perceived performance and actual performance. A second search was performed to obtain information on their content and psychometrics.

**Results:**

Regarding capacity, perceived performance and actual performance, 18, 9 and 3 instruments were included respectively. Only 3 of all included instruments were used and tested in both patient populations. The content of the instruments differed widely regarding the ICF levels measured, assessment of the amount of use versus the quality of use, the inclusion of unimanual and/or bimanual tasks and the inclusion of basic and/or extended tasks.

**Conclusions:**

Although many instruments assess capacity and perceived performance, a dearth exists of instruments assessing actual performance. In addition, instruments appropriate for more than one patient population are sparse. For actual performance, new instruments have to be developed, with specific focus on the usability in different patient populations and the assessment of quality of use as well as amount of use. Also, consensus about the choice and use of instruments within and across populations is needed.

## Background

Regarding arm-hand training, most treatment approaches target a specific patient population, and almost all arm-hand assessment instruments are currently applied in only one particular pathology. This leads to a myriad of patient-specific assessment tools. However, patients with different disorders with similar clinical characteristics might benefit from similar treatments, and assessment instruments might be suitable for more than one patient population. Dobkin [1] stated that the mechanisms of motor control, cognitive control and neural adaptation that accompany training and learning are not as much dependent on the underlying disease as on the spared nodes within neural networks. To enable a comparison of treatments within one patient population, and to gain more insight in the possibilities of common therapies for different patient populations with similar clinical characteristics, consensus should be reached regarding the choice and use of outcome measures that can be used across pathologies. Two disorders with similar clinical characteristics are stroke and cerebral palsy (CP). Whereas stroke is most prevalent in adults, CP is a neurologic disorder arising early in the development of children, i.e. during pregnancy, childbirth or early infancy [2]. Hemiparesis, spasticity and coordination disorders occur in both patients with stroke and patients with CP.

In stroke and CP, loss of arm-hand function and, consequently, loss of arm-hand performance leads to large problems in the everyday life of these patients. It limits the execution of activities of daily living, which results in greater dependency, restricted social participation [3], and a decreased quality of life [4]. After discharge from the hospital or rehabilitation centre, arm-hand function is often not fully recovered in patients with stroke [5] or CP [6]. Four years after stroke, 67% of the patients experience the non-use or disuse of the affected arm as a major problem, whereas only 6% of the patients is satisfied with their arm-hand function [7]. For CP, about 60% of the children between 4 and 16 years old have problems with their arm-hand function during daily pursuits [8,9].

Assessment of arm-hand function and performance is important in both clinical practice and research e.g. to determine the effectiveness of rehabilitation treatments and to monitor the progress of patients. In the present study, the term 'instrument' will refer to measurement instrument, i.e. instrument used to measure arm-hand function and/or performance.

**Figure 1:**
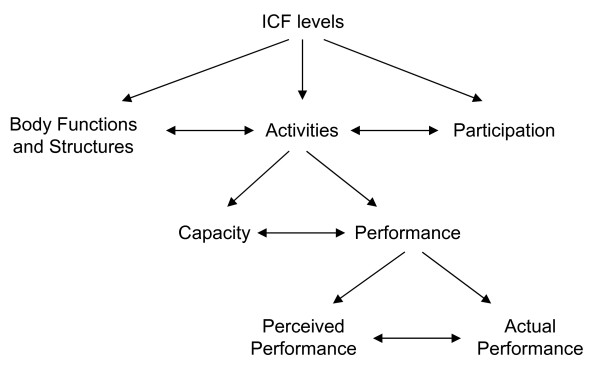
**ICF levels and subdivision**.

Next to its intended use, the International Classification of Functioning, Disability and Health (ICF) framework [10] can be used for the classification of outcome measures. The ICF describes human functioning at three levels (Figure 1), i.e. function level (body structures and function), activity level (task execution) and participation level (involvement in life situations). Activity level is subdivided into capacity and performance. The term arm-hand skilled performance (AHSP) used throughout this study, refers to arm-hand function at the ICF activity level [11], including both capacity and performance.

In the last decades, the focus of rehabilitation on arm-hand disorders is slowly shifting from ICF function level towards activity and participation level [12], i.e. towards a level that is important to the patient, whose typical question is "What will I be able to do with my arm and hand in my daily pursuits once therapy has finished?". However, the relationship between function level and activity level is still poorly understood. A study by Arnould et al. showed a relation between hand impairment (function level) and manual ability (activity level) in children with CP, but hand impairment predicted only 58% of the variability in manual ability measures [13]. Burridge et al. found significant correlations between several, though not all, measures at function level (e.g. active range of movement, and spasticity) and the Action Research Arm Test, a measure at activity level in patients with stroke [14].

Regarding activity level, it should be noted that outcomes of capacity measures and performance measures may differ strongly, since different constructs are measured, i.e. the highest level of functioning versus functioning in daily life situations. When assessing performance, like AHSP, two kinds of instruments are available. On the one hand, questionnaires can be used to measure perceived performance. On the other hand, actual performance can be measured by direct and objective assessment in the real-life situation.

For clinicians and researchers it is important to evaluate and compare the effects of therapies and treatments, in order to make a well-founded choice for the best therapy or treatment for the patient. This choice necessitates appropriate measurement instruments. Besides the differences in ICF levels and concepts (e.g. capacity or performance) instruments are quantifying, a large diversity in the content between instruments exists. To guide the choice of instruments, an overview of available instruments and their content is necessary. The aim of this systematic review is 1) to identify and evaluate the available instruments to assess AHSP in patients with stroke or CP and 2) to categorize the available instruments into the categories capacity, perceived performance and actual performance. Instruments for which no data about the validity and reliability was available were not included in this study.

## Methods

This review consists of 5 steps: 1) A systematic literature search was performed to identify articles which included instruments assessing AHSP in patients with stroke or CP. 2) Articles were selected based on predetermined inclusion criteria. 3) All measurement instruments reported within the articles selected were extracted. 4) Only instruments that fitted a second set of predetermined inclusion criteria were kept. All other instruments were discarded. 5) The included instruments were further evaluated and classified, yielding a final set of instruments that is reported on.

### Step 1: systematic literature search

A systematic search was performed to identify relevant articles published until November 2010, selected from the following databases: PubMed, CINAHL, EMBASE, Chochrane, PsychINFO, IEEE and Scopus. The search strategy consisted of 4 elements, focusing on 1) patient population; 2) upper extremity; 3) outcome domain and 4) measurement instrument. The combination of search terms used is listed in Table 1. Limits were set by using the terms 'human' and 'language: English, Dutch, German or French'.

**Table 1:** Search strategy

Patient population	"Stroke" NOT "stroke volume" OR "cerebral palsy" OR "motor skill disorder" OR "hemiparesis"
	AND

**Upper extremity**	("upper extremity" OR "upper limb" OR "arm" OR "hand" OR "wrist") NOT ("lower extremity" OR "lower limb")

	AND

**Outcome domain**	("motor activity" OR "activities of daily living" OR "motor skill" OR "motor skills" OR "function") NOT "gait"

	AND

**Measurement instrument**	("outcome assessment" OR "treatment outcome" OR "task performance and analysis" OR "evaluation studies as topics" OR "disability evaluations" OR "rehabilitation" OR questionnaires" OR "ambulatory monitoring") NOT ("magnetic resonance imaging" OR "blood" OR "cortex")

### Step 2: article selection

#### Inclusion and exclusion criteria

Articles were included if they met all of the following inclusion criteria: 1) the article should include instruments developed for and/or used in patients with stroke or CP; 2) the instrument used in the article should assess AHSP; 3) the article should be written in English, Dutch, German or French.

#### Screening of abstracts

All titles and abstracts of the articles retrieved from the literature search were divided among 4 reviewers and were screened for the inclusion and exclusion criteria. In cases where insufficient information was available from the title or the abstract, the full text of the article was examined. In cases of uncertainty, the title and abstract was screened by one of the other three reviewers. If it remained uncertain whether or not to include the article, the article was discussed between all 4 reviewers until consensus was reached. At the end of this step, a list of eligible articles was available.

#### Definitions

In this study several definitions have been used, adopted from the ICF [10]. In some cases ICF definitions were slightly adapted to adequately fit our needs in this review paper and to avoid misinterpretation. In literature, several terms and definitions exist to describe activities of daily living. No unambiguous definition exists to define basic ADL and extended ADL. Therefore, the definitions as described in Table 2 were formulated.

**Table 2:** Definitions

Term	Definition
Domain	A domain is a practical and meaningful set of related physiological functions, anatomical structures, actions, tasks, or areas of life.

Activity level	The level of execution of meaningful tasks by an individual.

Capacity	The highest possible level of functioning of a person in a given domain at a given moment, measured in a standardized environment.

Perceived performance	The level of functioning subjectively experienced by a person in a given domain at a given moment in his/her current environment.

Actual performance	The objectively detectable level of functioning of a person in a given domain at a given moment in his/her current environment.

Amount of use	How often (frequency) or how much (quantity) the arm-hand is used.

Quality of use	The quality with which the arm-hand is used during tasks or movements.

Unimanual tasks	Tasks which are usually performed with one hand.

Bimanual tasks	Tasks which are usually performed with both hands.

Activities of daily living (ADL)	Activities a person normally performs in daily life including activities performed for self-care, work, household activities and leisure.

Basic ADL	Activities of daily living necessary to daily self-care, including personal hygiene, dressing, feeding, toileting, functional transfers and mobility [15].

Extended ADL	Activities of daily living, beyond basic ADL, related to home maintenance and required for independent living. For example cleaning, cooking, doing laundry and shopping [15]

Classification instrument	Instrument used to describe upper limb performance on a level of categories, rather than to attribute scores that quantify upper limb performance.

### Step 3: data extraction: identification of instruments

The articles gathered from step 2 were analysed to identify instruments. Abstracts of these articles, and in case of insufficient information the full text, were screened to extract potential instruments. From several instruments more than one version was available. In such case, only 1 version of the instrument was included in this systematic review. The version which was tested on its validity and reliability in the target population and most often used was chosen.

### Step 4: inclusion of instruments

All instruments retrieved from step 3 were screened by 1 reviewer on the following inclusion and exclusion criteria. Inclusion criteria: 1) instrument assesses AHSP; 2) instrument has to be reported valid and reliable in the target population (stroke and/or CP). Exclusion criteria: 1) instrument is a classification instrument; 2) measuring AHSP is not the main goal of the instrument; 3) instrument is not uniquely used to asses AHSP.

Information about the properties and content of the instruments, necessary to apply the inclusion and exclusion criteria, were retrieved from several sources, i.e. from: 1) the articles included after the second step; 2) a new search on database Pubmed, using the name of the instrument (in combination with the following terms: 'valid' OR 'validity' OR 'reliable' OR 'reliability' OR 'responsive' OR 'responsiveness'); 3) other internet sources such as databases of instruments; 4) contact with authors describing the instruments. After this step, a final list of included instruments was composed.

### Step 5: classification and evaluation of instruments

The instruments from the final list were classified into the following categories: capacity, perceived performance and actual performance, based on the definitions presented earlier. In case of uncertainty about the classification, the instrument was discussed among the 4 reviewers, who are also experts in the field of arm-hand rehabilitation, until consensus was reached. The following information about each instrument was examined: target population, total number of items included in the test, number of items concerning measurement of the upper extremity, ICF levels measured with the instrument, inclusion of unimanual and/or bimanual items, assessment of amount of use and/or quality of use and the responsiveness.

## Results

**Figure 2:**
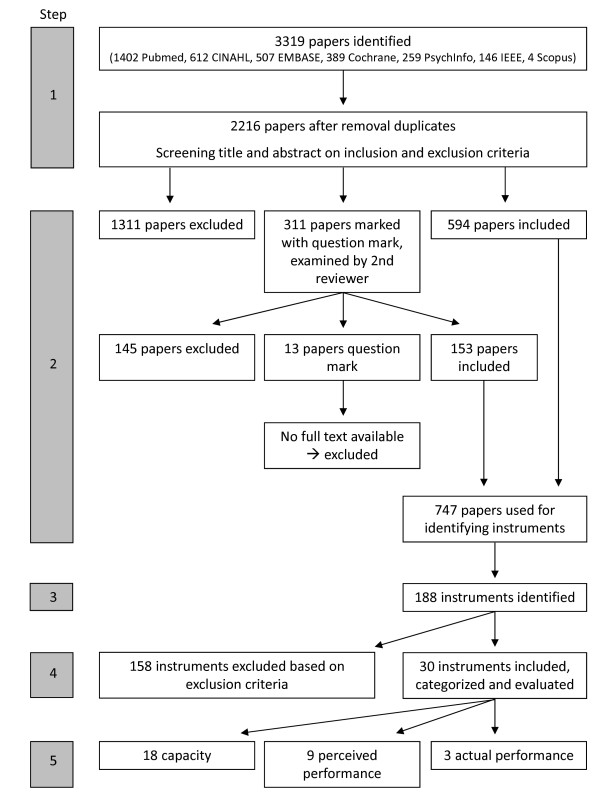
**Process of article selection and instrument selection**.

The systematic literature search (step 1) resulted in a total of 2216 articles, of which 747 were included after step 2. A total of 188 measurement instruments were identified from these articles (step 3) of which 30 were included (step 4), and further evaluated (step 5). A flowchart depicting this process is presented in Figure 2.

In Tables 3, 4 and 5 the instruments included in the categories capacity, perceived performance and actual performance are described, including information about the content of the instruments. Table 6 summarizes the number of instruments related to capacity, perceived performance and actual performance and the number and percentage of instruments per characteristic (e.g. target population, ICF level(s) measured, unimanual and/or bimanual tasks included, etc.).

**Table 3:** Instruments assessing capacity

Measurement instrument	Target population		Number of Items	ICF level(s) of the upper limb items			Tasks included		Activities of daily living included		Upper limb use			Reference		
	**Stroke**	**CP**		**Function**	**Activity**	**Participation**	**Unimanual**	**Bimanual**	**Basic**	**Extended**	**AOU**	**QOU**	**Other description of use^‡^**	**Validity**	**Reliability**	**Responsiveness**

	*Instruments including only items at ICF activity level*															

ADL observation [16]	√		4		√		(√)*	√	√	(√)*			√	[16]	[16]	

AMAT [17]	√		13		√		√	√	√	√		√	√	[17]	[17]	[17]

AMPS [15]	√	√	2		√			√	√*	√*		√		[15]	[15]	

CAHAI [18]	√		13		√			√	√	√			√	[19]	[19]	[19]

FAT [20]	√		5		√		√	√	√	√			√	[20]	[20]	[21]

TEMPA [22]	√		9		√		√	√	√	√		√	√	[23]	[22]	

UBDS [24]	√		1		√			√	√				√	[24]	[24]	[24]

VOAA-DDD [25]		√	2		√			√		√	√		√	[26]	[26]	

	*Instruments combining items at ICF activity level with items at ICF function level*															

AAUT [27]	√		17	√	√		√	√		√	√	√		[27]	[27]	

ARAT [28]	√		19	√	√		√			√		√		[28]	[28]	[28]

FTHUE [29]	√		17	√	√		√	√		√			√	[29]	[29]	

JTHFT [30]	√		7	√	√		√		√	√			√	[31]	[31]	[32]

Melbourne [33]		√	16	√	√		√		√	√		√		[33]	[34]	

MESUPES [35]	√		17	√	√		√			√		√	√	[35]	[35]	

MFT [36]	√		8	√	√		√			√			√	[37]	[37]	

QUEST [38]		√	36	√	√		√	√	√	√			√	[38]	[38]	

SHUEE [39]		√	40	√	√		√	√	√	√	√		√	[39]	[39]	

WMFT [40]	√		17	√	√		√			√			√	[41]	[41]	[41]

‡ = This subcategory covers answers like "can be performed (yes/no)" or "independent performance (yes/no/partly)"; or descriptive answers like "How much feedback does the patient require to complete the task?". * = item can be chosen by therapist/patient and can therefore be unimanual or bimanual and basic ADL or extended ADL.

CP = cerebral palsy; AOU = amount of use; QOU = quality of use.

AAUT = Actual Amount of Use Test; ADL observation = Activities of Daily Living observation; AMAT = Arm Motor Ability Test; AMPS = Assessment of Motor and Process Skills; ARAT = Action Research Arm Test; CAHAI = Chedoke Arm Hand Actvity Inventory; FAT = Frenchay Arm Test; FTHUE = Functional Test for the Hemiplegic Upper Extremity; JTHFT = Jebsen-Taylor Hand Function Test; LMAS = Lindmark Motor Assessment Scale; Melbourne = Melbourne Assessment of Unilateral Upper Limb Function; MESUPES = Motor Evaluation Scale for Upper Extremity Stroke Patients; MFT = Manual Function Test; QUEST = Quality of Upper extremity Skills Test; SHUEE = Shriners Hospital for Children Upper Extremity Evaluation; TEMPA = Upper Extremity Performance Test for Elderly (Test d'Evaluation des Membres supérieurs de Personnes Agées); UBDS = Upper Body Dressing Scale; VOAA-DDD = Video Observation Aarts and Aarts - DDD; WMFT = Wolf Motor Function Test

**Table 4:** Instruments assessing perceived performance

Measurement instrument	Target population		Number of Items	ICF level(s) of the upper limb items			Tasks included		Activities of daily living included		Upper limb use			Reference		
	**Stroke**	**CP**		**Function**	**Activity**	**Participation**	**Unimanual**	**Bimanual**	**Basic**	**Extended**	**AOU**	**QOU**	**Other description of use^‡^**	**Validity**	**Reliability**	**Responsiveness**

	*Instruments including only items at ICF activity level*															

Abilhand [42]	√		23		√		√	√	√	√			√	[43]	[43]	

Abilhand-kids [44]		√	21		√		√	√	√	√			√	[44]	[44]	

COPM [45]	√	√	variable		√		√*	√*	√*	√*			√	[46,47]	[46,47]	[47,48]

DHI [49]	√		18		√		√	√	√	√			√	[49]	[49]	[49]

GAS [50]	√	√	variable		√		√*	√*	√*	√*			√	[51,52]	[51,52]	[51,52]

HFS [53]	√		13		√		√	√	√	√			√	[53]	[53]	

MAL [54]	√		26		√		√	√	√	√	√	√		[54]	[54]	[54]

pMAL [55]		√	22		√		√	√	√	√	√	√		[56]	[56]	

	*Instruments combining items at ICF activity level with items at ICF function level*															

UE Item Bank [57]		√	49	√	√		√	√	√	√			√	[58,59]	[58]	

‡ = This subcategory covers answers like "can be performed (yes/no)" or "independent performance (yes/no/partly)"; or descriptive answers like "How much effort does it take to perform the task?". * = item can be chosen by therapist/patient and can therefore be unimanual or bimanual and basic ADL or extended ADL.

CP = cerebral palsy; AOU = amount of use; QOU = quality of use.

COPM = Canadian Occupational Performance Measure; DHI = Duruoz Hand Index; GAS = Goal Attainment Scaling; HFS = Hand Function Survey, MAL = Motor Activity Log; pMAL = Pediatric Motor Activity Log; UE Item bank = upper extremity item bank

**Table 5:** Instruments assessing actual performance


**Measurement instrument**	**Target population**		**Number of items**	**ICF level(s) of the upper limb items**			**Tasks included**		**Activities of daily living included**		**Upper limb use**			**Reference**		

	**Stroke**	**CP**		**Function**	**Activity**	**Participation**	**Unimanual**	**Bimanual**	**Basic**	**Extended**	**AOU**	**QOU**	**Other description of use^‡^**	**Validity**	**Reliability**	**Responsiveness**

Accelerometry [60]	√		Variable		√		√	√	√	√	√			[60]	[60]	

AHA [61]		√	Variable		√		√	√		√		√		[62]	[62]	[62]

FAABOS [63]	√		Variable		√		√	√	√	√	√			[63]	[63]	

‡ = This subcategory covers answers like "can be performed (yes/no)" or "independent performance (yes/no/partly)"; or descriptive answers like "How much effort does it take to perform the task?".

CP = cerebral palsy; AOU = amount of use; QOU = quality of use.

AHA = Assisting Hand Assessment; FAABOS = Functional Arm Activity Behavioral Observation System

**Table 6:** Overview of the number and percentage of instruments for different characteristics, presented per category

	Capacity		Perceived performance		Actual performance	
	**number**	**%**	**number**	**%**	**number**	**%**

**Total number measurement instruments**	18	100	9	100	3	100

**Target population**						

Stroke patients only	13	72.2%	4	44.4%	2	67%

CP patients only	4	22.2%	3	33.3%	1	33%

Stroke and CP	1	5.6%	2	22.2%	0	0%

**ICF level**						

Activity level only	8	44.4%	8	88.9%	3	100%

Activity & function level	10	55.6%	1	11.1%	0	0%

Activity & participation level	0	0%	0	0%	0	0%

Activity, function & participation level	0	0%	0	0%	0	0%

**Task included**						

Unimanual only	6	33.3%	0	0%	0	0%

Bimanual only	4	22.2%	0	0%	0	0%

Unimanual & bimanual	8	44.4%	9	100%	3	100%

**Activities of daily living included**						

Basic ADL only	1	5.6%	0	0%	0	0%

Extended ADL only	7	38.9%	0	0%	1	33%

Both basic and extended ADL	10	55.6%	9	100%	2	67%

**Upper limb use**						

AOU only	0	0%	0	0%	2	67%

QOU only	3	16.7%	0	0%	1	33%

Other description of use only	9	50%	7	77.8%	0	0%

Combination of AOU and QOU	1	5.6%	2	22.2%	0	0%

Combination of AOU and other description of use	2	11.1%	0	0%	0	0%

Combination of QOU and other description of use	3	16.7%	0	0%	0	0%

**Psychometric property**						

Responsiveness tested	7	38.8%	4	44.4%	1	33%

Responsiveness not tested	11	61.1%	5	55.6%	2	67%

CP = cerebral palsy; AOU = amount of use; QOU = quality of use; ADL = activities of daily living

The most noticeable difference is the number of instruments included per category. In the categories capacity and perceived performance, 18 and 9 instruments were included respectively, whereas only 3 instruments were categorized as measuring actual performance. In addition, more instruments are available for patients with stroke compared to patients with CP, with only 10% of the instruments used and tested for their psychometric properties in both patients groups. For 2 instruments, two versions exist, one for adults and one for children.

In the category capacity, about half of the instruments solely measure at ICF activity level. The other half of the instruments also measure at function level. In the category perceived performance almost 90% of the instruments solely measure at ICF activity level, whereas all instruments in the category actual performance measure only at ICF activity level.

In the category capacity, instruments consist of unimanual or bimanual items, or a combination of unimanual and bimanual items. In the category perceived performance, all instruments include both unimanual and bimanual items. In daily life, measured with instruments for actual performance, both unimanual and bimanual tasks are present. Most instruments include a combination of basic activities of daily living and extended activities of daily living.

Only a few instruments in the categories capacity and perceived performance measure quality of use (QOU), whereas no instruments measure the amount of use (AOU). Most instruments use another description to measure upper limb use for example how much assistance does the patient need to perform the task. Instruments assessing actual performance measure mostly AOU, but the Assisting Hand Aassessment (AHA) measures QOU of the affected arm-hand. For about half of all measurement instruments that were reported to be valid and reliable, responsiveness was also tested in the target population.

## Discussion

### Main findings

A total of 30 instruments were included in this review, 18 of which measure capacity, 9 measure perceived performance and 3 measure actual performance.

Even though stroke patients and cerebral palsy (CP) patients share most of their clinical symptoms, almost all of the upper extremity outcome measures are developed for, tested in and used in only one patient population, i.e. either stroke or CP. To gain more insight in the possibilities of common therapies, transcending diagnosis boundaries, agreement about the choice of common instruments is needed.

Regarding stroke or CP rehabilitation, many instruments are available in the categories capacity and perceived performance. However, instruments assessing actual performance are less abundantly available for these patients. This is in sharp contrast to the importance that patients, clinicians and researchers in the field of stroke and CP rehabilitation attribute to high quality arm-hand skilled performance (AHSP) in real life.

For capacity and perceived performance only a few instruments assess quality of use (QOU) of the affected arm and hand, whereas no instruments assess the amount of use (AOU). Many instruments use other descriptions of upper limb use, such as "how much assistance is needed to perform the task?".

In addition, this systematic review revealed that a large diversity in the content of the instruments exists, making it more difficult to compare different outcome measures with each other.

### Agreement about the choice and use of instruments

It can be concluded that a wide range of measurement instruments for the categories capacity and perceived performance exists. This systematic review identified 30 instruments currently available to assess AHSP in patients with stroke or CP, which are reported to be both valid and reliable. More than 155 instruments were excluded, mainly because no information was published about the psychometric properties in patients with stroke or CP. Other reasons for exclusion were for example "instrument does not include items to assess the upper extremity" and "instrument is a classification instrument". Nineteen instruments were excluded because next to arm-hand items, they contain also items not related to the upper extremity. Although instruments containing only arm-hand items are used most and are most appropriate to assess arm hand performance, the abovementioned nineteen instruments that were excluded might also be of interest in arm-hand assessment. For the sake of completeness, these instruments are listed in table 8 in the Additional file 1. The responsiveness has not been tested in about 60% of the instruments included in this review.

The chance of consistent use of outcome measures between studies decreases as the range of available instruments increases. The use of different outcome measures makes it more difficult to compare similar studies with each other. It is very important that a future agreement about the choice and the use of common instruments is achieved. This may facilitate comparison between studies, may result in more powerful meta-analyses, and enables the use of published data for group size calculations for new studies [64].

This systematic review demonstrated that only 3 out of 30 instruments were used in both patients with stroke and patients with CP (i.e. Assessment of Motor and Process Skills [15], the Goal Attainment Scale [50] and the Canadian Occupational Performance Measure [45]). In addition, for 2 instruments separate versions exist for adults and children (i.e. for the MAL/pMAL and the Abilhand/Abilhand-kids). Differences are for example age-dependent item content. Dobkin stated that the mechanisms of motor control, cognitive control and neural adaptation that accompany training and learning are not as much dependent on the underlying disease as on the spared nodes within neural networks [1]. Indeed it is seen that clinical practice paradigms to improve for instance arm-hand function do not tend to differ much between patient populations [1]. To gain more insight in the possibilities of common therapies for different patient populations with similar clinical characteristics, it is important that the same outcome measures are used. Only then a good comparison between studies assessing the same therapies, applied in different patient populations is possible and worthwhile.

It is important to investigate whether the outcome measure which can be used in several patient populations, is valid, reliable and responsive in each of the populations it is used in. One reason is that the course of improvement of AHSP, during and after rehabilitation, may differ between patient populations. Caused by, for example, the fact that stroke patients can rely on learned motor patterns which they have developed during their life, whereas in children with CP these motor patterns may not be present.

### Capacity and perceived performance

For the category capacity about 6 times more instruments are available than for the capacity actual performance. For the category perceived performance 3 times as many instruments are available.

Although information about the highest level of functioning (capacity) may be very useful, it does not reveal valid information about the functioning of a patient in daily life (performance). It is known that a large difference may exist between capacity and performance. This difference may be caused, among others, by the learned non-use phenomenon [27], developmental disregard [65], changes in the role of the patient at home and in the society [3] and the fact that capacity measures the highest possible level of functioning during a short period of time (i.e. time of testing) [10]. The latter does not mimic real life situations, where performance is continuous and, for instance, fatigue plays a role.

Patients, clinicians and researchers may have questions on both aspects of AHSP: capacity and performance. Depending on the information needed, the outcome measure should be chosen accordingly.

For the assessment of performance in stroke and CP, most instruments currently available evaluate perceived performance, whereas only 3 instruments assess actual performance. The questionnaires used to assess perceived performance take the perspective of the patient into account, which may be desirable but also has disadvantages. These questionnaires rely on recall and valid reporting of the patient. The cognitive problems stroke patients may have might influence the recall. In addition, the Hawthorn effect may play a role, i.e. the overestimation of arm-hand performance by the patient because his/her desire to improve or to please the examiner [66]. Furthermore, many children with CP are not able to fill in the questionnaire themselves and have to rely on parents and caregivers to fill in the questionnaire, leading to a different perspective, which may render the questionnaire invalid.

### Actual performance

Capacity and perceived performance are both relevant for the assessment of AHSP, but actual performance should equally be taken into account, since this reflects the real functioning of a patient in daily life. Actual performance is measured objectively. One example is video observation, in which the performance of a patient is unobtrusively monitored, while performing activities of daily living. A disadvantage of video observation is that the video material has to be assessed by (multiple) experts, which makes this method potentially subjective and very time consuming. Other disadvantages are the possible intrusion on the patient's privacy and the problems of installing the system in a patient's home.

Although the video-based AHA instrument is not applied in the home situation, it was classified as a measure for actual performance, because the spontaneous use of the affected arm during a 15 minutes free play is determined using video observation.

Another method to assess actual performance is accelerometry, measuring the actual AOU of the arm-hand in daily life. Wearing accelerometers is unobtrusive and data can be collected for several consecutive days. Because data collection is done during the whole day, the registered activity will include specific task-related movements, but also non-functional movements and unintentional arm activity. Accelerometry does not provide information about the QOU of the affected arm-hand. The latter is especially of interest for patients, clinicians and researchers. The QOU of the affected arm and hand is associated with the ability to use the affected arm and hand in the home situation, performing activities of daily living.

Currently several promising new instruments to assess actual performance have not yet been tested as to their psychometric properties and were therefore not included in this systematic review. Two examples of such instruments are the Strathclyde Upper Limb Activity Monitor (SULAM) [67] and the Stroke Upper Limb Activity Monitor (Stroke-ULAM) [68]. The SULAM uses a pressure transducer and electrohydraulic activity sensor to determine the vertical replacement of the wrist compared to the shoulder. The Stroke-ULAM consists of 5 accelerometers and 2 electrogoniometers, measuring the actual upper limb usage of both limbs and the percentage of activity of the affected limb compared to the unaffected limb.

In order to assess actual performance in stroke and CP, it is important that the systems under development will be tested more extensively to determine their utility and psychometric properties in both patient populations. In addition, measurement instruments for the assessment of actual performance have to be (further) developed, assessing also other aspects of AHSP such as QOU or information about the type of activity performed. Considering the importance of instruments transcending diagnosis boundaries, such instruments should be able to be used in different patient populations.

### Content of the instruments

A large diversity in the content of the instruments to assess AHSP in patients with stroke or CP exists. About half of the instruments included in the category capacity, solely measure at ICF activity level, whereas the other half of the instruments cover more ICF levels. About 89% of the instruments included in the category perceived performance and 100% of the instruments in the category actual performance solely measure at ICF activity level. If the aim of the study is to measure on ICF activity level, instruments assessing solely on ICF activity level are to be preferred. Whenever more ICF levels are included, the interpretation of the results becomes more difficult, especially when the outcome exists of a total score covering the different ICF levels.

The inclusion of unimanual and/or bimanual items differs among instruments. To determine the capacity of the affected arm-hand, unimanual tasks are useful because these tasks force the use of the affected arm-hand, which can be assessed. However, in daily life, many tasks are bimanual requiring both hands to perform the tasks. Moreover in daily life, the affected arm-hand is rarely used for unimanual tasks [69]. Therefore, if assessment of AHSP in daily life is aimed for, bimanual items should be included.

Although there are some differences in the inclusion of basic and/or extended activities of daily living, the majority of the instruments included both basic and extended activities of daily living.

### Considerations

Some considerations can be made regarding this systematic review. Based on the definitions stated earlier, some instruments which, in other studies, were classified as activity measures, were excluded in this systematic review, for example the nine hole peg test and box and block test [70]. However, the definitions were formulated in order to make a distinction between instruments including tasks which are meaningful in daily life and tasks which are not meaningful in daily life. Instruments containing activities of daily living in the items, but measure on function level (e.g. kinematics) were also excluded.

Instruments used as classification instruments rather than assessment tools for AHSP were excluded. Examples of such instruments are the Manual Ability Classification Instrument (MACS) [71] and the House classification [72].

Some instruments, such as the COPM and the GAS can be used to assess individual goals of patients. These instruments can be used to assess AHSP (whenever the individual goals are arm-hand activities) and have been demonstrated to be valuable in the assessment of AHSP [73]. Therefore, these instruments were included in this review, in contrast to other reviews [69,74]. This gives a more complete overview, of all instruments available. Moreover, individual goal setting instruments are valuable since they reflect the improvement of AHSP on the tasks which are most important for the patient.

### Limitations

This systematic review has some limitations that have to be addressed. One limitation might be the fact that the articles retrieved from the search strategy were divided among four reviewers. However, strict a priori rules were applied in the selection and evaluation of articles and instruments, and in case of even the slightest doubt, the article was reviewed by another reviewer and if needed discussed among all four reviewers.

A second limitation might be that in this systematic review, instruments were included whenever they were reported to be valid and reliable. No criteria were applied to determine the methodological quality of the studies describing the psychometrics. However, the aim of this review was to identify and evaluate instruments available for assessing AHSP in patients with stroke or CP, rather than to give an extended overview of the psychometric properties of these instruments. The latter one is an important next step.

## Conclusions

This systematic review provides an overview of available instruments to assess AHSP in patients with stroke and patients with CP. This overview may be used as a guide for instrument selection.

Currently, a limited number of valid and reliable instruments assessing actual performance for patients with stroke or CP are available. Furthermore, the 3 instruments available do not cover all domains interesting for AHSP. In order to assess actual performance, existing tools have to be adapted and new tools have to be developed, which will be applicable in more than one patient population. In addition, quantifying the QOU of the affected arm-hand should be taken into account while assessing activities of daily living.

Furthermore, only a few instruments are applicable for both patients with stroke and patients with CP. To better compare studies, and to gain more insight in the possibilities of common therapies for different patient populations with similar clinical characteristics, consensus should be reached regarding the choice and use of outcome measures which can be used in different patient populations.

## Funding

This paper was funded by Adelante, Centre of Expertise in Rehabilitation and Audiology, Hoensbroek, the Netherlands.

## Competing interests

The authors declare that they have no competing interests.

## Authors' contributions

RL participated in drafting the manuscript, carried out the literature search, participated in the screening of the articles, identified and evaluated the included instruments and participated in interpreting the results. AT, YJ and HS participated in drafting the manuscript, screening the articles and interpreting the results. Furthermore, AT, YJ and HS critically revised the intellectual content of the article. RS participated in drafting the manuscript and interpreting the results and critically revised the intellectual content of the article. All authors read and approved the final manuscript.

## Pre-publication history

The pre-publication history for this paper can be accessed here:

http://www.biomedcentral.com/1471-2377/12/21/prepub

## Supplementary Material

Additional file 1 **Excluded instruments **[75-203] Click here for file
