# Supplementary material for: Valid and reliable instruments for arm-hand assessment at ICF activity level in persons with hemiplegia: a systematic review
Source: BMC Neurol. 2012 Apr 12;12:21. doi: 10.1186/1471-2377-12-21 (PMC3352056; doi:10.1186/1471-2377-12-21)
Supplement: Additional file 1 — Excluded instruments [75-203] [file 1471-2377-12-21-S1.PDF]

Appendix

Table 7: List of excluded instruments.

**Instrument**
3 Activities of Daily Living [1]
3D motion analysis [2]
6 activities of Daily Living [3]
Ability to hold 1 kg [4]
Action Imitation Test [5]
Activities of Daily Living Index [6]
Activity Scale for Kids [7]
ADL Questionnaire [8]
ADL task performance [9]
Arm function Questionnaire [10]
Arm Function Test [11]
Australian therapy Outcome Measure for Occupational Therapy [12]
Barthel Index [13]
Bean spooning test [5]
Box and Block Test [14]
Bruininks-Oseretsky Test of Motor Proficiency [15]
Burke-Fahn-Marsden Scale [16]
California Functional Evaluation 40 [17]
Caregivers Functional Use Survey [18]
Chart review [19]
Child/Toddler Arm Use Test [20]
Daily Finger Feeding Task [21]
Dexterity Questionnaire for ADL [5]
Direct observation of ADL [22]
Disabilities of Arm Shoulder and Hand [23]
Disability Assessment Scale [24]
Drinking from a glass test [25]
Edinburg Handedness Inventory [26]
Emergency Behavior scale [20]
Emory Functon Test [27]
Erhardst Development Prehension assessment [14]
Fine motor speed [28]
Fitt's Tapping task [29]
fMRI [30]
Forward reaching distance [31]
Frenchay Activities Index [32]
Function evaluation score [25]

Function Test [33]
Functional Ability Scale [34]
Functional Activities [35]
Functional grips [36]
Functional Independence Measure [37]
Functional Independence Measure for Children [38]
Functional limitation profile [39]
Functional Tasks [25]
Grasp & displace cilinders test [40]
Grasp and lift a 600 gr weight [1]
Hand Function Test [41]
Haptic Rehabilitation Exercises Performance Evaluation [42]
House Classification [43]
In Hand Manipulation Test [44]
Index of Extended Activities of Daily Living [45]
Interview about ADL activities [22]
Inventory of New Motor Activities and Programs [46]
Jacket Test [47]
Katz Adjustement Index [45]
Katz Index [48]
Key Turning Task [49]
Keyboard abilities [50]
Kinematics [51]
Klein Bell Activities of Daily Living Scale [52]
Knox Parent Questionnaire [53]
Kocaeli Functional Evaluation Test [54]
Lawton Instrumental Activities of Daily Living [55]
Level of assisted required on 5 self-selected ADL tasks [9]
Lindmark Motor Assessment Scale [56]
Line tracing task [57]
Manual Ability Classification System [58]
Maximum Grip Force (on key) [49]
Meal preparation test [27]
Measurements of Activities of Daily Living [59]
Minnesota Handwriting Assessment [15]
Minnesota rate of manipulation test [25]
Motor Activity Log Actual Quality of Use [60]
Motor Activity Log grade 5 (low functioning patients) [61]
Motor Acvtivity Log Observation [62]
Motor Assessment Scale [63]
Motor Club Assessment [64]
Motor speed in grasping tasks (dexterous manipulation) [65]

Motor training task [9]
Motricity Index [66]
Movement assessment battery for children [67]
Movement speed task [25]
Mowery Classification [43]
Nine hole Peg test [66]
Northwick park dependency score [68]
Nottingham Extended Activities of Daily Living [69]
Nottingham health profile (part 1) [70]
Nottingham Leisure Questionnaire [45]
Nottinhham Stroke Dressing Assessment [71]
Nuts and Bolts Test [29]
Observation reach [72]
Ontario society of occupational therapists test [25]
Passive Function Task [73]
Patient Specific Function Scale [74]
Peabody Developmental Motor Scale [75]
Pediatric Arm Function Test [46]
Pediatric Evaluation of Disability Inventory [76]
Pediatric Outcome Data Collection Instrument [77]
Pediatric Quality of Life Inventory for children with Cerebral Palsy [78]
Pegboard Test [79]
Pendulum Test [80]
Pennie Test [22]
Perceived problems [10]
Performance Assessment of Self-care Skills [81]
Performance of 21 ADL skills (videotaped) [82]
Performance Quality Rating Scale [83]
Pig Tail Test [84]
Prehension Task [85]
Problem Score [86]
Purdue peg test [87]
Reaching Performance Scale [88]
Reaching Test [89]
Rehabilitatio Activities Profile [90]
Rehabilitation Engineering Laboratory Hand Function Test [91]
Rehabilitation Institute of Chigaco's Functional Assessment Scale [72]
Rivermead Activities of Daily Living Scale [92]
Rivermead Motor Assessment [93]
Rotation task [94]
Self Report Change [95]
Sensorimotor integration test battery [27]

Shift task [94]
Sickness Impact Profile 68 [96]
Simple Test for Evaluation Hand Function [97]
Sodring Motor Evaluation of Stroke patients [98]
Sollerman Hand Function Test [99]
Southern California Motor Accuracy Test [100]
Stacking blocks test [22]
Stacking cubes test [101]
Strathclyde Upper Limb Activity Monitor [102]
Stroke Adapted Sickness Impact Profile 30 [103]
Stroke Impact Scale [104]
Stroke Rehabilitation Assessment of Movement [105]
Stroke Upper Limb Activity Monitor [106]
Structures Assessment of Independent Living Skills [107]
Subjective comment [27]
Systematic Test for Assessing Reaching Times [108]
Task Performance test [109]
Ten hole Peg test [110]
Test Task [111]
The active lifestyle: efficacy expectancies Scale [45]
The Neurological Rehabilitation Score [112]
The Older American Resources and Services Scale: Activities of daily living [45]
Time to ADL Milestone [27]
Timed Task with Shoelace [29]
Transfer of coins [113]
Trowing coin in slot [114]
Two-hand functions [36]
University of Maryland Arm Questionnaire for Stroke [115]
Upper Extremity Function ADL Category [116]
Upper extremity functional index [25]
Upper Limb (Basic) Functional Activity Questionnaire [66]
Upper limb disability score [113]
Upper Limb Physicians Rating Scale [117]
Video-observation [43]
Vineland Adaptrive Behavior Scale [58]
Visual Analogue Scale for ADL [24]
Williams Doors [118]

Table 8: Excluded instruments which fulfil the inclusion criteria, but were excluded based on exclusion criteria 3 (instrument is not uniquely used to asses AHSP).

Measurement instrument	Target population		# Items upper limb/total	ICF level(s) of the upper limb items			Tasks included		Activities of daily living included		Upper limb use			Reference		
	Stroke	CP		Func-tion	Acti-vity	Partici-pation	Uni-manual	Bi-manual	Basic	Extended	AOU	QOU	Other descrip-tion of use‡	Vali-dity	Relia-bility	Respon-siveness
Capacity																
Instruments containing one or more subscale(s) with only items about the upper extremity																
	Instruments including only items at ICF activity level															
KB-ADL [52]	√		37/42		√		√	√	√	√			√	[52]	[52]	
	Instruments combining items at ICF activity level with items at ICF function level															
RMA [93]	√		18/38	√	√		√	√		√			√	[119]	[119]	[119]
SMES [98]	√		15/32	√	√		√	√	√			√		[120]	[121]	
Instruments containing partly items about the upper extremity, but with only one total score, without a subscale for the upper extremity																
	Instruments including only items at ICF activity level															
ADL Index [6]	√		10/17		√		√	√	√	√			√	[6]	[6]	
Katz index ADL [48]	√		4/6		√			√	√				√	[122]	[48]	
PASS [81]	√		18/26		√		√	√	√	√			√	[81]	[81]	
RADLS [92]	√		27/31		√		√	√	√	√			√	[92]	[92]	
	Instruments combining items at ICF activity level with items at ICF function level															
MAS [63]	√		3/9	√	√		√	√	√	√			√	[123]	[63]	[124]

NSDA [71]	√		Male:10/15<br>Female: 13/18	√	√			√	√			√	[71]	[71]	
Perceived Performance															
Instruments containing one or more subscale(s) with items only about the upper extremity															
	Instruments including only items at ICF activity level														
FIM [37]	√		6/18		√		√	√	√			√	[125]	[125]	[125]
WeeFIM [38]		√	6/18		√		√	√	√			√	[126]	[126]	[127]
	Instruments combining items at ICF activity level with items at ICF function level and/or participation level														
AusTOMs-OT [12]	√		5/12	√	√	√	√	√	√	√		√	[128]	[12]	[12]
FAI [32]	√		13/15		√	√		√		√	√		[129]	[130]	[129]
NEADL [69]	√		17/22		√	√		√	√	√		√	[129]	[69]	[129]
Peds-QL-CP[78]		√	16/35	√	√		√	√	√	√		√	[78]	[78]	[78]
SA-SIP 30 [103]	√		7/30	√	√		√	√	√	√		√	[103]	[103]	
SIS [104]	√		19/65	√	√	√	√	√	√	√		√	[104]	[104]	[104]
Instruments containing partly items about the upper extremity, but with only one total score, without a subscale for the upper extremity															
	Instruments including only items at ICF activity level														
BI [13]	√		5/10		√			√	√			√	[13]	[13]	[13]
	Instruments combining items at ICF activity level with items at ICF function level and/or participation level														
RAP [90]	√		8/21		√	√		√	√	√		√	[131]	[132]	[133]

‡ = This subcategory covers answers like “can be performed (yes/no)” or “independent performance (yes/no/partly)”; or descriptive answers like “How much effort does it take to perform the task?”.

CP = cerebral palsy; AOU = Amount of use; QOU = Quality of Use.

ADL Index = Activities of Daily Living Index; AusTOMs-OT = Australian therapy Outcome Measure for Occupational Therapy; BI = Barthel Index; FAI = Frenchay Activities Index; FIM = Functional Independence Measure; Katz Index ADL = Katz index of Independence in Activities of Daily Living; KB-ADL = Klein Bell Activities of Daily Living Scale; MAS = Motor Assessment Scale; NEADL = Nottingham Extended Activities of Daily Living; NSDA = Nottingham Stroke Dressing Assessment; PASS = Performance Assessment of Self-care Skills; PedsQL-CP = Pediatric Quality of Life Inventory for children with Cerebral Palsy; RADLS = Rivermead Activities of Daily Living Scale; RAP = Rehabilitation Activities Profile; RMA = Rivermead Motor Assessment; SA-SIP 30 = Stroke Adapted Sickness Impact Profile 30; SIS = Stroke Impact Scale; SMES = Sodring Motor Evaluation of Stroke patients; WeeFIM = Functional Independence Measure for Children.

References of excluded instruments

1. Alon G, McBride K, Ring H: **Improving selected hand functions using a noninvasive neuroprosthesis in persons with chronic stroke**. *Journal of Stroke & Cerebrovascular Diseases* 2002, **11**(2):99-106.
2. Woldag H, Hummelsheim H: **Is the reduction of spasticity by botulinum toxin a beneficial for the recovery of motor function of arm and hand in stroke patients?** *Eur Neurol* 2003, **50**(3):165-171.
3. Huber M, Rabin B, Docan C, Burdea G, Nwosu ME, Abdelbaky M, Golomb MR: **PlayStation 3-based tele-rehabilitation for children with hemiplegia**. In *Virtual Rehabilitation, 2008: 25-27 Aug. 2008 2008*; 2008:105-112.
4. Alon G, Dar A, Katz-Behiri D, Weingarden H, Nathan R: **Efficacy of a hybrid upper limb neuromuscular electrical stimulation system in lessening selected impairments and dysfunctions consequent to cerebral damage**. *Journal of Neurologic Rehabilitation* 1998, **12**(2):73-79.
5. Sunderland A: **Recovery of ipsilateral dexterity after stroke**. *Stroke* 2000, **31**(2):430-433.
6. Sheikh K, Smith DS, Meade TW, Goldenberg E, Brennan PJ, Kinsella G: **Repeatability and validity of a modified activities of daily living (ADL) index in studies of chronic disability**. *Int Rehabil Med* 1979, **1**(2):51-58.
7. Postans N, Wright P, Bromwich W, Wilkinson I, Farmer SE, Swain I: **The combined effect of Dynamic splinting and Neuromuscular electrical stimulation in reducing wrist and elbow contractures in six children with Cerebral palsy**. *Prosthet Orthot Int*, **34**(1):10-19.

8. Staubli P, Nef T, Klamroth-Marganska V, Riener R: **Effects of intensive arm training with the rehabilitation robot ARMin II in chronic stroke patients: four single-cases**. *J Neuroeng Rehabil* 2009, **6**:46.

9. McEwen SE, Huijbregts MP, Ryan JD, Polatajko HJ: **Cognitive strategy use to enhance motor skill acquisition post-stroke: a critical review**. *Brain Inj* 2009, **23**(4):263-277.

10. Broeks JG, Lankhorst GJ, Rumping K, Prevo AHJ: **The long-term outcome of arm function after stroke: results of a follow-up study**. *Disability & Rehabilitation* 1999, **21**(8):357-364.

11. Wade DT, Langton-Hewer R, Wood VA, Skilbeck CE, Ismail HM: **The hemiplegic arm after stroke: measurement and recovery**. *J Neurol Neurosurg Psychiatry* 1983, **46**(6):521-524.

12. Unsworth CA: **Measuring Outcomes using the Australian Therapy Outcome Measures for Occupational Therapy (AusTOMs-OT): Data Description and Tool Sensitivity**. *British Journal of Occupational Therapy* 2005, **68**(8):354-366.

13. Quinn TJ, Langhorne P, Stott DJ: **Barthel index for stroke trials: development, properties, and application**. *Stroke*, **42**(4):1146-1151.

14. Sung I, Ryu J, Pyun S, Yoo S, Song W, Park M: **Efficacy of forced-use therapy in hemiplegic cerebral palsy**. *Archives of Physical Medicine & Rehabilitation* 2005, **86**(11):2195-2198.

15. Bumin G, Kavak ST: **An investigation of the factors affecting handwriting skill in children with hemiplegic cerebral palsy**. *Disabil Rehabil*, **32**(8):692-703.

16. Smania N, Gambarin M, Tinazzi M, Picelli A, Fiaschi A, Moretto G, Bovi P, Paolucci S: **Are indexes of arm recovery related to daily life autonomy in patients with stroke?** *Eur J Phys Rehabil Med* 2009, **45**(3):349-354.

17. Byl N, Roderick J, Mohamed O, Hanny M, Kotler J, Smith A, Tang M, Abrams G: **Effectiveness of sensory and motor rehabilitation of the upper limb following the principles of neuroplasticity: patients stable poststroke**. *Neurorehabil Neural Repair* 2003, **17**(3):176-191.

18. Charles JR, Wolf SL, Schneider JA, Gordon AM: **Efficacy of a child-friendly form of constraint-induced movement therapy in hemiplegic cerebral palsy: a randomized control trial**. *Dev Med Child Neurol* 2006, **48**(8):635-642.

19. Spaulding SJ, Strachota E, McPherson JJ, Kuphal M, Ramponi M: **Wrist muscle tone and self-care skill in persons with hemiparesis**. *Am J Occup Ther* 1989, **43**(1):11-16.

20. Taub E, Ramey SL, DeLuca S, Echols K: **Efficacy of constraint-induced movement therapy for children with cerebral palsy with asymmetric motor impairment**. *Pediatrics* 2004, **113**(2):305-312.

21. Crocker MD, MacKay-Lyons M, McDonnell E: **Forced use of the upper extremity in cerebral palsy: a single-case design**. *Am J Occup Ther* 1997, **51**(10):824-833.

22. Kinghorn J: **Upper extremity functional changes following selective posterior rhizotomy in children with cerebral palsy**. *Am J Occup Ther* 1992, **46**(6):502-507.

23. Karabegovic A, Kapidzic-Durakovic S, Ljuca F: **Laser therapy of painful shoulder and shoulder-hand syndrome in treatment of patients after the stroke**. *Bosn J Basic Med Sci* 2009, **9**(1):59-65.

24. Van Heest AE, Strothman D: **Wrist arthrodesis in cerebral palsy**. *J Hand Surg Am* 2009, **34**(7):1216-1224.

25. van Dijk H, Jannink MJ, Hermens HJ: **Effect of augmented feedback on motor function of the affected upper extremity in rehabilitation patients: a systematic review of randomized controlled trials**. *J Rehabil Med* 2005, **37**(4):202-211.

26. Coderre AM, Zeid AA, Dukelow SP, Demmer MJ, Moore KD, Demers MJ, Bretzke H, Herter TM, Glasgow JI, Norman KE *et al*: **Assessment of upper-limb sensorimotor function of subacute stroke patients using visually guided reaching**. *Neurorehabilitation & Neural Repair*, **24**(6):528-541.

27. van der Lee JH, Snels IA, Beckerman H, Lankhorst GJ, Wagenaar RC, Bouter LM: **Exercise therapy for arm function in stroke patients: a systematic review of randomized controlled trials**. *Clin Rehabil* 2001, **15**(1):20-31.

28. Størvold GV, Jahnsen R: **Intensive motor skills training program combining group and individual sessions for children with cerebral palsy**. *Pediatric Physical Therapy*, **22**(2):150-159.

29. Turton AJ, Fraser CM: **A test battery to measure the recovery of voluntary movement control following stroke**. *Int Rehabil Med* 1986, **8**(2):74-78.

30. Takahashi CD, Der-Yeghiaian L, Le V, Motiwala RR, Cramer SC: **Robot-based hand motor therapy after stroke**.

31. Chan MK, Tong RK, Chung KY: **Bilateral upper limb training with functional electric stimulation in patients with chronic stroke**. *Neurorehabil Neural Repair* 2009, **23**(4):357-365.

32. Holbrook M, Skilbeck CE: **An activities index for use with stroke patients**. *Age Ageing* 1983, **12**(2):166-170.

33. Smania N, Aglioti SM, Cosentino A, Camin M, Gandolfi M, Tinazzi M, Fiaschi A, Faccioli S: **A modified constraint-induced movement therapy (CIT) program improves paretic arm use and function in children with cerebral palsy**. *Eur J Phys Rehabil Med* 2009, **45**(4):493-500.

34. Wolf SL, Thompson PA, Morris DM, Rose DK, Winstein CJ, Taub E, Giuliani C, Pearson SL: **The EXCITE trial: attributes of the Wolf Motor Function Test in patients with subacute stroke**. *Neurorehabil Neural Repair* 2005, **19**(3):194-205.

35. Kinghorn J, Roberts G: **The effect of an inhibitive weight-bearing splint on tone and function: a single-case study**. *Am J Occup Ther* 1996, **50**(10):807-815.

36. Autti-Ramo I, Larsen A, Peltonen J, Taimo A, von Wendt L: **Botulinum toxin injection as an adjunct when planning hand surgery in children with spastic hemiplegia**. *Neuropediatrics* 2000, **31**(1):4-8.

37. Keith RA, Granger CV, Hamilton BB, Sherwin FS: **The functional independence measure: a new tool for rehabilitation**. *Adv Clin Rehabil* 1987, **1**:6-18.

38. Msall ME, DiGaudio K, Rogers BT, LaForest S, Catanzaro NL, Campbell J, Wilczenski F, Duffy LC: **The Functional Independence Measure for Children (WeeFIM). Conceptual basis and pilot use in children with developmental disabilities**. *Clin Pediatr (Phila)* 1994, **33**(7):421-430.

39. Ietswaart M, Johnston M, Dijkerman HC, Scott CL, Joice SA, Hamilton S, Macwalter RS: **Recovery of hand function through mental practice: a study protocol**. *BMC Neurol* 2006, **6**:39.

40. Steenbergen B, Verrel J, Gordon AM: **Motor planning in congenital hemiplegia**. *Disabil Rehabil* 2007, **29**(1):13-23.

41. Yozbatiran N, Donmez B, Kayak N, Bozan O: **Electrical stimulation of wrist and fingers for sensory and functional recovery in acute hemiplegia**. *Clin Rehabil* 2006, **20**(1):4-11.

42. Barghout A, Alamri A, Eid M, El Saddik A: **Haptic Rehabilitation Exercises Performance Evaluation Using Fuzzy Inference Systems**. In *Medical*

Measurements and Applications, 2008 MeMeA 2008 IEEE International Workshop on: 9-10 May 2008 2008; 2008:13-18.

43. Waters PM, Zurakowski D, Patterson P, Bae DS, Nimec D: **Interobserver and intraobserver reliability of therapist-assisted videotaped evaluations of upper-limb hemiplegia**. *J Hand Surg Am* 2004, **29**(2):328-334.

44. Breslin DM, Exner CE: **Construct validity of the In-Hand Manipulation Test: a discriminant analysis with children without disability and children with spastic diplegia**. *Am J Occup Ther* 1999, **53**(4):381-386.

45. **Therapy-based rehabilitation services for stroke patients at home**. *Cochrane Database Syst Rev* 2003(1):CD002925.

46. Taub E, Griffin A, Nick J, Gammons K, Uswatte G, Law CR: **Pediatric CI therapy for stroke-induced hemiparesis in young children**. *Dev Neurorehabil* 2007, **10**(1):3-18.

47. Watson MJ, Crosby P, Matthews M: **An evaluation of the effects of a dynamic lycra orthosis on arm function in a late stage patient with acquired brain injury**. *Brain Inj* 2007, **21**(7):753-761.

48. Brorsson B, Asberg KH: **Katz index of independence in ADL. Reliability and validity in short-term care**. *Scand J Rehabil Med* 1984, **16**(3):125-132.

49. Alberts JL, Butler AJ, Wolf SL: **The effects of constraint-induced therapy on precision grip: a preliminary study**. *Neurorehabil Neural Repair* 2004, **18**(4):250-258.

50. Steenbergen B, Veringa A, de Haan A, Hulstijn W: **Manual dexterity and keyboard use in spastic hemiparesis: a comparison between the impaired hand and the 'good' hand on a number of performance measures**. *Clin Rehabil* 1998, **12**(1):64-72.

51. Schneiberg S, McKinley P, Gisel E, Sveistrup H, Levin MF: **Reliability of kinematic measures of functional reaching in children with cerebral palsy**. *Dev Med Child Neurol*, **52**(7):e167-173.

52. Klein RM, Bell BJ: *Klein-Bell Activities of Daily Living Scale*.

53. Cope SM, Forst HC, Bibis D, Liu XC: **Modified constraint-induced movement therapy for a 12-month-old child with hemiplegia: a case report**. *Am J Occup Ther* 2008, **62**(4):430-437.

54. Dursun N, Dursun E, Sade I, Cekmece C: **Constraint induced movement therapy: efficacy in a Turkish stroke patient population and evaluation by a new outcome measurement tool**. *Eur J Phys Rehabil Med* 2009, **45**(2):165-170.

55. Carod-Artal FJ, Coral LF, Trizotto DS, Moreira CM: **The stroke impact scale 3.0: evaluation of acceptability, reliability, and validity of the Brazilian version**. *Stroke* 2008, **39**(9):2477-2484.

56. Welmer AK, Holmqvist LW, Sommerfeld DK: **Limited fine hand use after stroke and its association with other disabilities**. *J Rehabil Med* 2008, **40**(8):603-608.

57. Yoo E, Park E, Chung B: **Mental practice effect on line-tracing accuracy in persons with hemiparetic stroke: a preliminary study**. *Arch Phys Med Rehabil* 2001, **82**(9):1213-1218.

58. van Eck M, Dallmeijer AJ, van Lith IS, Voorman JM, Becher J: **Manual ability and its relationship with daily activities in adolescents with cerebral palsy**. *J Rehabil Med*, **42**(5):493-498.

59. Blank R, von Kries R, Hesse S, von Voss H: **Conductive education for children with cerebral palsy: effects on hand motor functions relevant to activities of daily living**. *Arch Phys Med Rehabil* 2008, **89**(2):251-259.

60. Sterr A, Freivogel S, Schmalohr D: **Neurobehavioral aspects of recovery: assessment of the learned nonuse phenomenon in hemiparetic adolescents**. *Arch Phys Med Rehabil* 2002, **83**(12):1726-1731.

61. Bowman MH, Taub E, Uswatte G, Delgado A, Bryson C, Morris DM, McKay S, Mark VW: **A treatment for a chronic stroke patient with a plegic hand combining CI therapy with conventional rehabilitation procedures: case report**. *NeuroRehabilitation* 2006, **21**(2):167-176.

62. Weber DJ, Skidmore ER, Niyonkuru C, Chang CL, Huber LM, Munin MC: **Cyclic functional electrical stimulation does not enhance gains in hand grasp function when used as an adjunct to onabotulinumtoxinA and task practice therapy: a single-blind, randomized controlled pilot study**. *Arch Phys Med Rehabil*, **91**(5):679-686.

63. Carr JH, Shepherd RB, Nordholm L, Lynne D: **Investigation of a new motor assessment scale for stroke patients**. *Phys Ther* 1985, **65**(2):175-180.

64. Sunderland A, Tinson D, Bradley L, Hewer RL: **Arm function after stroke. An evaluation of grip strength as a measure of recovery and a prognostic indicator**. *J Neurol Neurosurg Psychiatry* 1989, **52**(11):1267-1272.

65. Santos M, Zahner LH, McKiernan BJ, Mahnken JD, Quaney B: **Neuromuscular electrical stimulation improves severe hand dysfunction for individuals with chronic stroke: a pilot study**. *J Neurol Phys Ther* 2006, **30**(4):175-183.

66. Shaw L, Rodgers H, Price C, van Wijck F, Shackley P, Steen N, Barnes M, Ford G, Graham L: **BoTULS: a multicentre randomised controlled trial to evaluate the clinical effectiveness and cost-effectiveness of treating upper limb spasticity due to stroke with botulinum toxin type A**. *Health Technol Assess*, **14**(26):1-113, iii-iv.

67. Radell U, Tillberg E, Mattsson E, Amark P: **Postnatal cerebral infection leading to hemiplegic cerebral palsy: functional limitations and disability of 13 children in Sweden**. *Disabil Rehabil* 2008, **30**(25):1910-1919.

68. Bhakta B, Cozens A, Farrin A, Ford G, Langan D, McCabe C, et al.: **DARS. Dopamine Augmented Rehabilitation in Stroke. Does Co-careldopa treatment in combination with routine NHS occupational and physical therapy, delivered early after stroke within a stroke service, improve functional recovery including walking and arm function?** *http://wwwemeacuk/projectfiles/084361protocolpdf*.

69. Nouri FM, Lincoln NB: **An extended activities of daily living scale for stroke patients**. *Clin Rehabil* 1987, **1**(4):301-305.

70. Kwakkel G, Wagenaar RC: **Dynamics of functional recovery after cerebrovascular attack: effects of intensity of training of hand and walking skills [Dutch]**. *Nederlands Tijdschrift Voor Fysiotherapie* 2000, **110**(2):32-40.

71. Fletcher-Smith J, Walker M, Sunderland A, Garvey K, Wan A, Turner H: **An interrater reliability study of the Nottingham Stroke Dressing Assessment**. *British Journal of Occupational Therapy* 2010, **73**(12):570-578.

72. Lewin JE, Mix CM, Gaebler-Spira D: **Self-help and upper extremity changes in 36 children with cerebral palsy subsequent to selective posterior rhizotomy and intensive occupational and physical therapy**. *Physical & Occupational Therapy in Pediatrics* 1993, **13**(3):25-42.

73. Ashford S, Turner-Stokes L: **Management of shoulder and proximal upper limb spasticity using botulinum toxin and concurrent therapy interventions: a preliminary analysis of goals and outcomes**. *Disabil Rehabil* 2009, **31**(3):220-226.

74. Siebers A, Ã–berg U, Skargren E: **Improvement and impact of initial motor skill after intensive rehabilitation - CI-therapy in patients with chronic hemiplegia. A follow-up study**. *Advances in Physiotherapy* 2006, **8**(4):146-153.

75. Olesch CA, Greaves S, Imms C, Reid SM, Graham HK: **Repeat botulinum toxin-A injections in the upper limb of children with hemiplegia: a randomized controlled trial**. *Dev Med Child Neurol*, **52**(1):79-86.

76. Wesdock KA, Kott K, Sharps C: **Pre- and postsurgical evaluation of hand function in hemiplegic cerebral palsy: exemplar cases**. *J Hand Ther* 2008, **21**(4):386-397.

77. Barnes D, Linton JL, Sullivan E, Bagley A, Oeffinger D, Abel M, Damiano D, Gorton G, Nicholson D, Romness M *et al*: **Pediatric outcomes data collection instrument scores in ambulatory children with cerebral palsy: an analysis by age groups and severity level**. *J Pediatr Orthop* 2008, **28**(1):97-102.

78. Varni JW, Burwinkle TM, Berrin SJ, Sherman SA, Artavia K, Malcarne VL, Chambers HG: **The PedsQL in pediatric cerebral palsy: reliability, validity, and sensitivity of the Generic Core Scales and Cerebral Palsy Module**. *Dev Med Child Neurol* 2006, **48**(6):442-449.

79. Burtner PA, Poole JL, Torres T, Medora AM, Abeyta R, Keene J, Qualls C: **Effect of wrist hand splints on grip, pinch, manual dexterity, and muscle activation in children with spastic hemiplegia: a preliminary study**. *J Hand Ther* 2008, **21**(1):36-42; quiz 43.

80. de Kroon JR, Ijzerman MJ, Chae J, Lankhorst GJ, Zilvold G: **Relation between stimulation characteristics and clinical outcome in studies using electrical stimulation to improve motor control of the upper extremity in stroke**. *J Rehabil Med* 2005, **37**(2):65-74.

81. Skidmore ER, Rogers JC, Chandler LS, Holm MB: **Dynamic interactions between impairment and activity after stroke: examining the utility of decision analysis methods**. *Clin Rehabil* 2006, **20**(6):523-535.

82. Wolf SL, Lecraw DE, Barton LA, Jann BB: **Forced use of hemiplegic upper extremities to reverse the effect of learned nonuse among chronic stroke and head-injured patients**. *Exp Neurol* 1989, **104**(2):125-132.

83. McEwen SE, Polatajko HJ, Huijbregts MPJ, Ryan JD: **Exploring a cognitive-based treatment approach to improve motor-based skill performance in chronic stroke: results of three single case experiments**. *Brain Injury* 2009, **23**(13-14):1041-1053.

84. Yelnik A, Bonan I, Debray M, Lo E, Gelbert F, Bussell B: **Changes in the execution of a complex manual task after ipsilateral ischemic cerebral hemispheric stroke**. *Archives of Physical Medicine & Rehabilitation* 1996, **77**(8):806-810.

85. Boian R, Sharma A, Han C, Merians A, Burdea G, Adamovich S, Recce M, Tremaine M, Poizner H: **Virtual reality-based post-stroke hand rehabilitation**. *Stud Health Technol Inform* 2002, **85**:64-70.

86. van der Lee JH, Wagenaar RC, Lankhorst GJ, Vogelaar TW, Devillé WL, Bouter LM: **Forced use of the upper extremity in chronic stroke patients: results from a single-blind randomized clinical trial**. *Stroke; a journal of cerebral circulation* 1999, **30**(11):2369-2375.

87. Schaechter JD, Perdue KL: **Enhanced cortical activation in the contralesional hemisphere of chronic stroke patients in response to motor skill challenge**.

88. Stoykov ME: **Bilateral training for upper extremity hemiparesis in stroke**. *Ph.D.* University of Illinois at Chicago, Health Sciences Center; 2008.

89. Hiraoka K: **Rehabilitation effort to improve upper extremity function in post-stroke patients: a meta-analysis**. *Journal of Physical Therapy Science* 2001, **13**(1):5-9.

90. Van Bennekom CA, Jelles F, Lankhorst GJ: **Rehabilitation Activities Profile: the ICIDH as a framework for a problem-oriented assessment method in rehabilitation medicine**. *Disabil Rehabil* 1995, **17**(3-4):169-175.

91. Thrasher TA, Zivanovic V, McIlroy W, Popovic MR: **Rehabilitation of reaching and grasping function in severe hemiplegic patients using functional electrical stimulation therapy**. *Neurorehabil Neural Repair* 2008, **22**(6):706-714.

92. Lincoln NB, Edmans JA: **A re-validation of the Rivermead ADL scale for elderly patients with stroke**. *Age Ageing* 1990, **19**(1):19-24.

93. Lincoln N, Leadbitter D: **Assessment of motor function in stroke patients**. *Physiotherapy* 1979, **65**(2):48-51.

94. Eliasson AC, Bonnier B, Krumlinde-Sundholm L: **'Clinical experience of constraint induced movement therapy in adolescents with hemiplegic cerebral palsy--a day camp model'**. *Dev Med Child Neurol* 2003, **45**(5):357-359.

95. Almeida GL, Campbell SK, Girolami GL, Penn RD, Corcos DM: **Multidimensional assessment of motor function in a child with cerebral palsy following intrathecal administration of baclofen**. *Phys Ther* 1997, **77**(7):751-764.

96. de Groot-Driessen D, van Heugten C: **Speed of finger tapping as a predictor of functional outcome after unilateral stroke**. *Archives of Physical Medicine & Rehabilitation* 2006, **87**(1):40-44.

97. Kawahira K, Shimodozono M, Etoh S, Kamada K, Noma T, Tanaka N: **Effects of intensive repetition of a new facilitation technique on motor functional recovery of the hemiplegic upper limb and hand**. *Brain Injury*, **24**(10):1202-1213.

98. Sodring KM, Bautz-Holter E, Ljunggren AE, Wyller TB: **Description and validation of a test of motor function and activities in stroke patients. The Sodring Motor Evaluation of Stroke Patients**. *Scand J Rehabil Med* 1995, **27**(4):211-217.

99. Brogardh C, SjÃ¶lund BH: **Constraint-induced movement therapy in patients with stroke: a pilot study on effects of small group training and of extended mitt use**. *Clinical Rehabilitation* 2006, **20**(3):218-227.

100. Talbot ML, Junkala J: **The effects of auditorally augmented feedback on the eye-hand coordination of students with cerebral palsy**. *Am J Occup Ther* 1981, **35**(8):525-528.

101. Wolf TM, Clinkscales CM, Hamlin C: **Flexor carpi ulnaris tendon transfers in cerebral palsy**. *J Hand Surg Br* 1998, **23**(3):340-343.

102. Vega-Gonzalez A, Bain BJ, Dall PM, Granat MH: **Continuous monitoring of upper-limb activity in a free-living environment: a validation study**. *Med Biol Eng Comput* 2007, **45**(10):947-956.

103. van Straten A, de Haan RJ, Limburg M, Schuling J, Bossuyt PM, van den Bos GA: **A stroke-adapted 30-item version of the Sickness Impact Profile to assess quality of life (SA-SIP30)**. *Stroke* 1997, **28**(11):2155-2161.

104. Duncan PW, Wallace D, Lai SM, Johnson D, Embretson S, Laster LJ: **The stroke impact scale version 2.0. Evaluation of reliability, validity, and sensitivity to change**. *Stroke* 1999, **30**(10):2131-2140.

105. Sullivan JE, Hedman LD: **A home program of sensory and neuromuscular electrical stimulation with upper-limb task practice in a patient 5 years after a stroke**. *Phys Ther* 2004, **84**(11):1045-1054.

106. de Niet M, Bussmann JB, Ribbers GM, Stam HJ: **The stroke upper-limb activity monitor: its sensitivity to measure hemiplegic upper-limb activity during daily life**. *Arch Phys Med Rehabil* 2007, **88**(9):1121-1126.

107. Henderson A, Korner-Bitensky N, Levin M: **Virtual reality in stroke rehabilitation: a systematic review of its effectiveness for upper limb motor recovery**. *Top Stroke Rehabil* 2007, **14**(2):52-61.

108. Duff M, Attygalle S, He J, Rikakis T: **A portable, low-cost assessment device for reaching times**. *Conf Proc IEEE Eng Med Biol Soc* 2008, **2008**:4150-4153.

109. Lewis GN, Byblow WD: **Neurophysiological and behavioral adaptations to a bilateral training intervention in individuals following stroke**.

110. Walker CM, Sunderland A, Sharma J, Walker MF: **The impact of cognitive impairment on upper body dressing difficulties after stroke: a video analysis of patterns of recovery**. *J Neurol Neurosurg Psychiatry* 2004, **75**(1):43-48.

111. Sheppard L, Mudie H, Froude E: **An investigation of bilateral isokinematic training and neurodevelopmental therapy in improving use of the affected hand in children with hemiplegia**. *Physical & Occupational Therapy in Pediatrics* 2007, **27**(1):5-25.

112. Thilmann A, Nachtmann A, Scharff A: **[Neurological Reha-Score. An instrument to measure outcome and expenditure of neurologic rehabilitation]**. *Nervenarzt* 2006, **77**(12):1456-1463.

113. Reeuwijk A, van Schie PE, Becher JG, Kwakkel G: **Effects of botulinum toxin type A on upper limb function in children with cerebral palsy: a systematic review**. *Clin Rehabil* 2006, **20**(5):375-387.

114. Shah SK, Corones J: **Volition following hemiplegia**. *Arch Phys Med Rehabil* 1980, **61**(11):523-528.

115. Waller SM, Whitall J: **Hand dominance and side of stroke affect rehabilitation in chronic stroke**. *Clinical Rehabilitation* 2005, **19**(5):544-551.

116. Yagura H, Miyai I, Seike Y, Suzuki T, Yanagihara T: **Benefit of inpatient multidisciplinary rehabilitation up to 1 year after stroke**. *Arch Phys Med Rehabil* 2003, **84**(11):1687-1691.

117. Yang TF, Fu CP, Kao NT, Chan RC, Chen SJ: **Effect of botulinum toxin type A on cerebral palsy with upper limb spasticity**. *Am J Phys Med Rehabil* 2003, **82**(4):284-289.

118. Sunderland A, Bowers MP, Sluman S, Wilcock DJ, Ardron ME: **Impaired dexterity of the ipsilateral hand after stroke and the relationship to cognitive deficit**. *Stroke (00392499)* 1999, **30**(5):949-955.

119. Kurtais Y, Kucukdeveci A, Elhan A, Yilmaz A, Kalli T, Tur BS, Tennant A: **Psychometric properties of the Rivermead Motor Assessment: its utility in stroke**. *J Rehabil Med* 2009, **41**(13):1055-1061.

120. Wyller TB, Sodring KM, Sveen U, Ljunggren AE, Bautz-Holter E: **Predictive validity of the Sodring Motor Evaluation of Stroke Patients (SMES)**. *Scand J Rehabil Med* 1996, **28**(4):211-216.

121. Halsaa KE, Sodring KM, Bjelland E, Finsrud K, Bautz-Holter E: **Inter-rater reliability of the Sodring Motor Evaluation of Stroke patients (SMES)**. *Scand J Rehabil Med* 1999, **31**(4):240-243.

122. Asberg KH, Nydevik I: **Early prognosis of stroke outcome by means of Katz Index of activities of daily living**. *Scand J Rehabil Med* 1991, **23**(4):187-191.

123. Malouin F, Pichard L, Bonneau C, Durand A, Corriveau D: **Evaluating motor recovery early after stroke: comparison of the Fugl-Meyer Assessment and the Motor Assessment Scale**. *Arch Phys Med Rehabil* 1994, **75**(11):1206-1212.

124. English CK, Hillier SL, Stiller K, Warden-Flood A: **The sensitivity of three commonly used outcome measures to detect change amongst patients receiving inpatient rehabilitation following stroke**. *Clin Rehabil* 2006, **20**(1):52-55.

125. Dodds TA, Martin DP, Stolov WC, Deyo RA: **A validation of the functional independence measurement and its performance among rehabilitation inpatients**. *Arch Phys Med Rehabil* 1993, **74**(5):531-536.

126. Tur BS, Kucukdeveci AA, Kutlay S, Yavuzer G, Elhan AH, Tennant A: **Psychometric properties of the WeeFIM in children with cerebral palsy in Turkey**. *Dev Med Child Neurol* 2009, **51**(9):732-738.

127. Ottenbacher KJ, Msall ME, Lyon N, Duffy LC, Ziviani J, Granger CV, Braun S, Feidler RC: **The WeeFIM instrument: its utility in detecting change in children with developmental disabilities**. *Arch Phys Med Rehabil* 2000, **81**(10):1317-1326.

128. Unsworth CA, Duckett SJ, Duncombe D, Perry A, Skeat J, Taylor N: **Validity of the AusTOM scales: a comparison of the AusTOMs and EuroQol-5D**. *Health Qual Life Outcomes* 2004, **2**:64.

129. Wu CY, Chuang LL, Lin KC, Horng YS: **Responsiveness and validity of two outcome measures of instrumental activities of daily living in stroke survivors receiving rehabilitative therapies**. *Clin Rehabil*, **25**(2):175-183.

130. Post MW, de Witte LP: **Good inter-rater reliability of the Frenchay Activities Index in stroke patients**. *Clin Rehabil* 2003, **17**(5):548-552.

131. van Bennekom CA, Jelles F, Lankhorst GJ, Bouter LM: **The Rehabilitation Activities Profile: a validation study of its use as a disability index with stroke patients**. *Arch Phys Med Rehabil* 1995, **76**(6):501-507.

132. Jelles F, Van Bennekom CA, Lankhorst GJ, Sibbel CJ, Bouter LM: **Inter- and intra-rater agreement of the Rehabilitation Activities Profile**. *J Clin Epidemiol* 1995, **48**(3):407-416.

133. van Bennekom CA, Jelles F, Lankhorst GJ, Bouter LM: **Responsiveness of the rehabilitation activities profile and the Barthel index**. *J Clin Epidemiol* 1996, **49**(1):39-44.
